# Nationwide Seroprevalence of *Coxiella burnetii* Infection in Saudi Farm Animals: Implications for Public Health

**DOI:** 10.3390/vetsci12070629

**Published:** 2025-07-01

**Authors:** Samy Kasem, Roua A. Alsubki, Ahmed Saad, Kamal H. Zidan, Ibrahim Qasim, Osman Hashim, Ali Alkarar, Ali Abu-Obeida, Eman Damra, Zaaima Al-Jabri, Ahmed S. Abdel-Moneim, Waleed Al-Salem

**Affiliations:** 1 Department of Virology, Faculty of Veterinary Medicine, Kafrelsheikh University, El Geish Street, Kafrelsheikh 33516, Egypt; 2 National Livestock and Fisheries Development Program, Ministry of Water, Environment and Agriculture, Riyadh 4925-12361, Saudi Arabia; 3 Department of Clinical Laboratory Science, College of Applied Medical Science, King Saud University, Riyadh 11451, Saudi Arabia; 4 Ministry of Environment, Water and Agriculture, 65 King Abdulaziz Road, Riyadh 11195, Saudi Arabia; 5 Department of Microbiology and Immunology, College of Medicine and Health Sciences, Sultan Qaboos University, Muscat 123, Oman

**Keywords:** Q fever, ELISA, IgG, seroprevalence, livestock, Saudi Arabia

## Abstract

Q fever, caused by the intracellular bacterium *Coxiella burnetii*, is a significant zoonotic disease for which ruminants are the main reservoir. This cross-sectional study aimed to assess the seroprevalence of *C. burnetii* in farm animals (sheep, goats, cattle, and camels) across Saudi Arabia. A total of 7760 serum samples were collected from 2253 sheep, 2224 goats, 1111 cattle, and 2172 camels, representing various regions of the country. The samples were screened for *C. burnetii* antibodies using an enzyme-linked immunosorbent assay (ELISA). The findings revealed significant regional and species-specific differences. The findings revealed notable regional and species-specific variations in seroprevalence. In goats, seropositivity was detected in 92% of the tested herds; however, only 48% of the individually tested animals were found to be positive. Similarly, camels exhibited herd-level seropositivity of 92.9% of the examined herds, with only 46.7% of the individually examined animals testing positive. For sheep, 80% of the examined sheep flocks were positive, while 30.2% of the individually tested animals were positive. Cattle showed a significantly lower seroprevalence, since only 27.6% of the screened herds were found to be positive, and only 8.2% of the individually tested animals were positive. In conclusion, the results indicate that *C. burnetii* infection is widespread among livestock in Saudi Arabia, with goats, camels, and sheep posing a particularly elevated risk of zoonotic transmission. The observed regional disparities and species-specific infection rates highlight the need for comprehensive surveillance and targeted control strategies to mitigate the spread of Q fever in Saudi Arabia.

## Simple Summary

Q fever, a disease caused by the bacterium *Coxiella burnetii*, affects both animals and humans. This national survey conducted across Saudi Arabia aimed to assess the prevalence of Q fever among farm animals. The findings revealed high infection rates in goats, sheep, and camels, while cattle showed significantly lower rates of infection. The infection levels also varied by region, with certain areas reporting a greater number of cases. These results demonstrate the importance of strengthening disease surveillance and implementing effective control measures to help prevent the spread of Q fever to humans.

## 1. Introduction

Q fever, caused by the intracellular bacterium *Coxiella burnetii*, is a zoonotic disease that affects both animals and humans. It belongs to the γ subgroup of proteobacteria [1,2]. *C. burnetii* is a globally distributed, Gram-negative, obligate intracellular bacterium, while Q fever is a zoonotic disease that affects a wide range of animal species, including ruminants, equines, canines, felines, non-human primates, game, and marine mammals, as well as fish, amphibians, and reptiles [3,4,5]. Ticks are considered a potential, though not primary, vector of *C. burnetii*, playing a role in its transmission to wild and, occasionally, domestic animals [3]. Domesticated species, including dairy cattle, sheep, and goats, as well as camels, are recognized reservoirs of Q fever and may act as sources of infection for humans [6,7].

Airborne transmission of *C. burnetti* is the principal route for infection in both animals and humans, while contaminated clinical materials and unpasteurized milk have been identified as additional sources [8]. Contaminated dust and aerosols from *C. burnetii*-positive farms are considered some of the primary sources of human infections [9,10].

In animals, this microorganism is primarily found in the reproductive system, where it can cause both miscarriage and infertility. In humans, infection with *C. burnetii* typically presents as an acute flu-like illness, hepatitis, pneumonia, or chronic endocarditis. This disease affects different age groups in various ways and is more frequent in men than women, with mortality from chronic forms ranging between 1 and 11% [11].

*C. burnetii* DNA can be detected in animals using a PCR test and a wide range of clinical materials, including vaginal secretions, abortion products, feces, milk, urine, and blood. This method has gained popularity in diagnostic laboratories equipped with PCR capabilities [12,13]. In the 1960s, the first serological evidence of Q fever was reported in Saudi Arabia among Riyadh’s inhabitants [14]; however, the fever was not documented in animals until 2008 [15]. In 2012, antibodies against the pathogen were reported in Saudi wildlife [16]. Recently, *C. burnetii* DNA was detected in clinical specimens from camels, goats, cattle, and sheep in Saudi Arabia [17]. In addition, *C. burnetii*-specific IgG antibodies were detected at King Khalid University Hospital, Riyadh, in 18 (35.2%) out of 51 human patients who had a history of febrile illness lasting between four and eight weeks. Moreover, two of fifty healthy controls were found to harbor *C. burnetii*-specific IgG antibodies at titers sufficient to be considered positive [18]. Since studies have identified *C. burnetii* as a potential cause in patients with pyrexia of unknown origin (PUO), the surveillance and control of Q fever in livestock is of particular importance.

While comprehensive data on the seroprevalence of Q fever across all animal species in Saudi Arabia remain limited, this study provides the first nationwide assessment in key domestic livestock (sheep, goats, cattle, and camels). These findings aim to assist health policymakers establish and modify control and prevention programs for Q fever to mitigate zoonotic risks in Saudi Arabia.

## 2. Materials and Methods

### 2.1. Study Design

**Figure 1:**
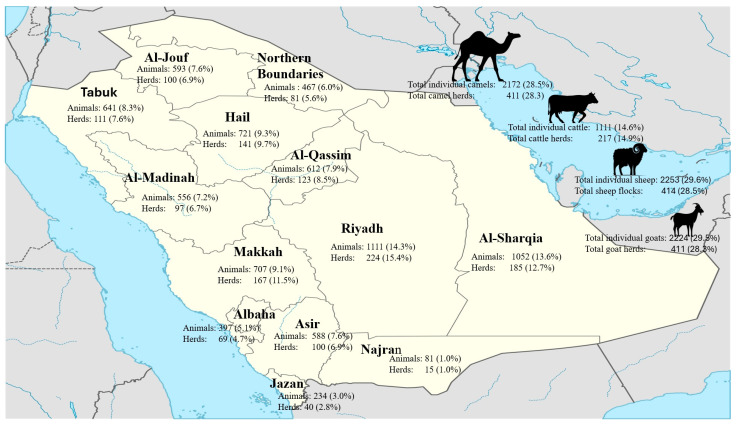
Number of animals examined from different regions of Saudi Arabia. For each area, the number of herds tested and individual samples collected is shown. The total number tested for each animal species (both in terms of individuals and herds) is displayed on the right side of the map.

The study design was conducted in accordance with the ethical approval granted by the Scientific Committee of the Animal Sector, Ministry of Environment, Water, and Agriculture, in March 2017 [06-1438]. This national cross-sectional survey was carried out by the Saudi Ministry of the Environment, Water, and Agriculture (MEWA), Riyadh, Saudi Arabia, as part of a national survey on the seroprevalence of *C. burnetii.* The survey was conducted during the period from May to July 2017 and covered all 13 administrative regions of Saudi Arabia. A total of 7760 serum samples were collected from 1453 herds, including 2253 samples from 414 sheep flocks, 2224 samples from 411 goat herds, 1111 samples from 217 cattle herds, and 2172 samples from 411 camel herds (Figure 1).

Herds were selected using a stratified random sampling approach, where the strata were defined by animal species and administrative region. Within each stratum, herds were randomly selected in proportion to the livestock population size and density reported by MEWA in the most recent national census. This study sampled 414 sheep flocks (3.5% of the national flocks), 411 goat herds (5.9%), 217 cattle herds (4.1%), and 411 camel herds (20.5%).

Within each selected herd, 5–10 animals were randomly sampled depending on herd size and species. The within-herd sample size was calculated to achieve 95% confidence in detecting at least one seropositive animal if the true prevalence was ≥10%, assuming an intra-herd prevalence of at least 30% and adjusting for design effect.

All the tested animals showed no clinical symptoms of the disease, appeared to be healthy, and were treated in accordance with the animal welfare code of Saudi Arabia.

Blood samples (5–10 mL) were collected from each animal via jugular venipuncture, with the volume adjusted for species size. Age and sex data of animals were not collected due to logistical and field limitations associated with the scale and scope of sampling. Sera were separated by centrifugation at 3000 rpm for 10 min and stored at –20 °C until further testing.

### 2.2. Serological Assay

Commercial Q fever antibody indirect multi-species ELISA kits (IDvet, Montpellier, France) were used to detect anti-*Coxiella burnetii* IgG antibodies in the serum samples, following the manufacturer’s instructions. The microwells provided were plates pre-coated with a *C. burnetii* antigen derived from a bovine isolate. The test and control serum samples were diluted to a final dilution of 1:50 using the provided dilution buffer before being applied to the wells. Each test run included negative control, and positive controls Biochrom Ltdto ensure the assay’s validity. After the incubation and washing steps, an anti-multi-species horseradish peroxidase (HRP) conjugate was added. Subsequently, 100 µL of the substrate solution containing TMB (3,3′,5,5′-tetramethylbenzidine) was added and incubated in the dark at room temperature for 15 min. The colorimetric reaction was then stopped by adding 100 µL of the stop solution (0.5 M sulfuric acid), and the optical density (OD) was measured at 450 nm using a microplate ELISA reader (Asys Expert Plus, Waterbeach, Cambridge, United Kingdom) within 10 min of stopping the reaction.

The results were analyzed using IDSoft® software version 5.05, as recommended by the manufacturer. Based on the software’s interpretation criteria, samples with a Sample/Positive (S/P) ratio above 50% were considered positive, those with a ratio between >40% and <50% were considered doubtful, and values ≤ 40% were classified as negative. The S/P ratio was calculated as follows: (OD_sample − OD_negative_control)/(OD_positive_control − OD_negative_control) × 100. According to the manufacturer’s specifications, the assay demonstrated 100% sensitivity (95% CI: 89.28–100%) and 100% specificity (95% CI: 97.75–100%).

### 2.3. Data Management and Statistical Analysis

The collected data were transferred into a Microsoft Excel spreadsheet and then imported into the Statistical Package for Social Sciences (SPSS) for Windows® Version 22.0 (SPSS Inc., Chicago, IL, USA) for statistical analysis appropriate to each variable. The analyses were performed using a 2-tailed chi-square test and logistic regression model. The associations found using the chi-square test and logistic regression model were considered significant when *p* ≤ 0.05.

## 3. Results

### 3.1. Prevalence of Seropositivity

*C. burnetii* antibodies were detected in dairy cattle and camels across all surveyed regions, with varying prevalence rates. Specific immunoglobulins against *C. burnetii* were identified through an ELISA in 2853 (36.8%) out of the 7760 samples collected from a total of 1453 herds. The herds that showed positive results included 331/414 (80%) sheep flocks, 378/411 (92%) goat herds, 382/411 (92.8%) camel herds, and 60/217 (27.6%) cattle herds (Table 1).

### 3.2. Q Fever Seroprevalence in Sheep

The seroprevalence of Q fever antibodies in sheep across various regions of Saudi Arabia was assessed, with the prevalence rates examined at both the individual animal and herd levels. The overall herd-level prevalence was 80% (331 out of the 414 herds tested). The highest herd prevalence was recorded in Al-Jouf, where 96% of the herds tested positive for Q fever antibodies, followed by Al-Sharqia (87.3%) and Al-Qassim (87.3%). Despite a high herd-level prevalence, significant variations were observed in the individual animal-level seroprevalence, suggesting complex infection dynamics within these herds.

For example, in the Hail region, 73.3% of the herds tested positive, yet only 42.8% of the individual animals were found to be seropositive. In contrast, the Northern Boundaries region demonstrated a higher herd-level prevalence (87.3%) but a notably lower individual animal-level seroprevalence (20%). Similarly, in regions such as Riyadh, Al-Sharqia, and Al-Jouf, where the herd-level prevalence was relatively high (81.4%, 87.3%, and 96%, respectively), the individual animal-level seroprevalence rates varied, with values of 29.7%, 34.2%, and 34.2%, respectively. In Jazan, Makkah, and Al-Madinah, all the tested herds (100%) were positive for Q fever, indicating high herd-level exposure in these regions, but the individual animal-level seroprevalence varied from 17.2% in Najran to 54.2% in Al-Madinah, further illustrating the complex infection dynamics. The regional variations in the prevalence were statistically significant, with a highly significant result for the individual animal-level seroprevalence (*p* = 0.000), while the herd-level prevalence showed a less significant difference (*p* = 0.07) (Table 1).

### 3.3. Q Fever Seroprevalence in Goats

The overall herd-level seroprevalence of *C. burnetii* was 92% (378 out of the 411 herds tested), indicating a widespread exposure to *C. burnetii* across the country. The highest herd seroprevalence was reported in Jazan, Al-Madinah, and Makkah, where all the herds (100%) tested positive for Q fever antibodies, suggesting a high level of circulation in these regions. However, despite 100% herd-level positivity, individual animal-level seroprevalence varied, reflecting heterogeneous transmission. For example, while in the Northern Boundaries region, 84% of the herds were seropositive, only 23.9% of individual animals tested positive. In the Hail region, 91% of the herds were positive, with 67.2% of the tested animals being positive. In Jazan, Makkah, and Al-Madinah, all the tested herds (100%) were positive for Q fever; however, 49.1%, 54.2%, and 44.2% of the individually screened animals were found to be positive for the infection, respectively. The regional variations in the herd-level prevalence were statistically significant, as indicated by the chi-square test, which yielded a highly significant result (*p* = 0.005) (Table 1).

### 3.4. Q Fever Seroprevalence in Cattle

The positive individual animal-level seroprevalence was 8.2% (91 out of the 1111 cattle tested), while the herd-level seroprevalence was 27.6% (60 out of the 217 herds tested). The highest herd-level prevalence was recorded in Al-Madinah, where 57.1% of the herds tested positive, followed by Riyadh (43.5%) and Makkah (43.8%). These regions also showed a notable individual animal-level seroprevalence, with 17.9% recorded in Riyadh, 14.2% in Makkah, and 15.2% in Al-Madinah. Significant regional variations were observed in both measures of the seroprevalence, with a chi-square test demonstrating highly significant differences in both the individual animal- (*p* < 0.001) and herd-level (*p* = 0.0014) prevalence, indicating that regional differences in both the herd exposure and individual animal infection rates were statistically significant. For instance, in the Hail, Al-Qassim, and Northern Boundaries regions, no seropositive herds or individual animals were recorded, indicating that the cattle in these regions had no exposure to *C. burnetii*. At the individual animal level, the highest seroprevalence was found in Riyadh (17.9%), followed by Makkah (14.2%) and Al-Madinah (15.2%). In contrast, regions such as Al-Sharqia, Al-Qassim, and the Northern Boundaries showed a minimal or negligible seroprevalence (2.5%, 3.3%, and 2.8%, respectively) (Table 1).

### 3.5. Q Fever Seroprevalence in Camels

The individual animal-level seroprevalence was 46.7% (1014 out of the 2172 camels tested), and the herd-level prevalence was 92.9% (382 out of the 411 herds tested). The highest herd-level prevalence was observed in Riyadh, where 95% of the herds tested positive, followed closely by regions such as Hail, Al-Qassim, and Makkah, with 93.3%, 97.2%, and 97.8% positive herds, respectively. These regions also showed a relatively high individual animal-level seroprevalence, with Hail having the highest at 76.6%, followed by Al-Qassim (69.4%) and Tabuk (61.7%). On the other hand, regions such as Makkah, Jazan, and Najran demonstrated a lower individual animal-level seroprevalence, with Makkah showing the lowest at 18.8%. However, Makkah still had a high herd-level prevalence (97.8%). Statistically, regional differences in individual-level prevalence were highly significant (*p* < 0.001), while herd-level differences were not statistically significant (Table 1).

## 4. Discussion

This study provides a comprehensive analysis of the seroprevalence of *C. burnetii*, the causative agent of Q fever, across various domestic livestock species in Saudi Arabia, including sheep, goats, camels, and cattle. Using an ELISA, we detected antibodies against *C. burnetii* in 2853 (36.8%) out of the 7760 collected samples. These results highlight significant, widespread infections across different species, which is consistent with the findings of similar studies conducted globally [19,20,21,22,23,24].

For sheep, seroprevalence was positive in 30.2% of the tested samples, with a remarkably high flock-level prevalence as 80% of the examined flocks testing positive. The highest flock-level prevalence was recorded in Al-Jouf (96%), followed by Al-Sharqia and Al-Qassim (87.3%). The variation in the individual animal-level seroprevalence between regions, exemplified by the relatively low rate of 42.8% in Hail despite a high herd-level prevalence and the very low rate of 20% in the Northern Boundaries region, reflects the complexity of infection dynamics at the individual level. The global relevance of these findings is further supported by similar reports from other countries, where sheep have been identified as a significant host for *C. burnetii* and a source of zoonotic outbreaks [25,26]. In Saudi Arabia, regions like Jazan, Makkah, and Al-Madinah reported a 100% herd-level seroprevalence, which suggests widespread exposure within these areas. This finding further emphasizes the potential risk of human Q fever transmission from sheep, particularly in regions where herds are densely populated.

For goats, seroprevalence was 48% for individually examined animals, with antibodies detected in 92% of the screened herds. These results align with findings from other regions, where the seroprevalence in goats has been determined to range from 13% to 60.6%, emphasizing the role goats play as a major source of *C. burnetii* infection for humans [22,23,27]. Our results also demonstrated considerable regional variation, with the highest seroprevalence found in Hail (67.2%) and the lowest in the Northern Boundaries region (23.9%). The high seroprevalence of *C. burnetii* in goats, especially in areas like Hail, suggests that these animals likely contribute to the zoonotic transmission of the pathogen in Saudi Arabia.

In camels, the proportion of seropositive herds was notably high (92.9%). In Makkah, although this region showed the highest herd-level seroprevalence rates (97.8%), only 18% of the animals tested within the herds here were found to be seropositive. Herds in some regions, such as Hail, Al-Qassim, and Tabuk, showed significantly high herd exposure (93.3% to 97.2%) and had the highest proportion of individually seropositive animals (61.7% to 76.6%). This finding reflects a complex infection pattern, where a high amount of herd-level seropositivity to *C. burnetii* does not always align with a high amount of individual-level seropositivity. Camels are known to shed *C. burnetii* through their milk, urine, feces, and blood. Their high shedding potential, combined with the common practice of consuming raw camel milk, highlights their role in transmitting the pathogen to humans [7,17].

In contrast, cattle exhibited the lowest amount of herd-level seropositivity to *C. burnetii* (27.6%) of all the screened species, with only 8.2% of individually tested animals showing positive results. While cattle may contribute to the transmission of *C. burnetii*, particularly via birthing products such as placentas and birth fluids [28], their lower amount of exposure is likely due to differences in farming practices that influence the spread of *C. burnetii.* For example, the higher amount of seroprevalence detected in goats, camels, and sheep could be attributed to the fact that these animals are kept in larger, more open environments with less biosecurity. In contrast, cattle are often kept in more controlled, confined environments that reduce their exposure to this pathogen. Studies from other countries, including Kenya, Nigeria, Spain, and Italy, have reported similar low cattle seroprevalence rates [19,20,27,29].

Our study also revealed statistically significant differences in the seroprevalence of individual animals in different regions and among the different animal species tested, suggesting that local factors, such as climate conditions and co-grazing with other species, significantly influence the transmission of *C. burnetii.* These findings are consistent with studies that have highlighted the role of shared grazing environments in facilitating the spread of Q fever [21].

Reproductive cycles and environmental factors likely play a major role in the spread of *Coxiella burnetii*. The bacterium is shed in large amounts during birth, especially in placenta, amniotic fluid, and vaginal secretions. This makes the period around parturition a high-risk time for transmission [28]. In animals like sheep and goats, which often give birth around the same time of year, this can lead to concentrated environmental contamination and increased exposure within herds or flocks [26]. Windy and dry environmental conditions may also help spread contaminated dust over larger areas, raising the risk of infection for nearby animals and humans [10]. These seasonal and environmental factors may help explain the differences in seroprevalence observed between regions.

This nationwide cross-sectional study provided critical insights into the *C. burnetii* seroprevalence in Saudi livestock, though several design aspects warrant discussion. While our stratified random sampling of 7760 animals from 1453 herds ensured the representation of a variety of regions and species, the absence of age-stratified data limited our ability to distinguish recent infections from historical exposure, a known constraint of serological surveys [29,30]. This study relied on ELISA to detect antibodies but could not confirm active shedding or quantify the transmission risks, a limitation often seen in Q fever research [28,31]. Notably, our sampling frame may have underrepresented nomadic herds, which constitute ~30% of Saudi livestock [17] and could exhibit distinct exposure patterns due to their mobility.

The interpretation of our seroprevalence results must also consider the diagnostic performance of the ELISA used. While the commercial multi-species ELISA employed in this study is widely used for detecting *C. burnetii* antibodies and is reported by the manufacturer to have high sensitivity and specificity, its performance can vary across animal species and infection stages. A recent evaluation by Lurier et al. (2021) using latent class models [32] demonstrated notable differences in the diagnostic accuracy of three commercial ELISA kits when applied to cattle, sheep, and goats, with sensitivity and specificity estimates showing considerable variability [32]. These differences may result in underestimation or overestimation of the true prevalence, particularly when used in large-scale surveillance studies without confirmatory testing. Therefore, our findings should be interpreted in the context of potential diagnostic limitations, and future work may benefit from combining serological assays with molecular diagnostics such as PCR to enhance accuracy.

Moreover, strain-specific genetic diversity of *C. burnetii* may also play a significant role in the observed variation in seroprevalence across species and regions. Different ruminant hosts may be infected with distinct genotypes, which can influence host specificity, pathogenicity, and shedding potential. For example, three main genotype clusters of *C. burnetii* were identified in French livestock, with strong species-specific associations and geographic stability, suggesting ecological and epidemiological segregation among host species [33]. Similarly, genotype heterogeneity was reported in northern Spain, indicating that some genotypes are more frequently associated with goats, while others are predominant in cattle or sheep [34]. A phylogeographic study from Belgium also highlighted the coexistence of multiple *C. burnetii* strains in both human and animal populations, underscoring the potential for zoonotic transmission linked to specific genotypes [35]. These findings suggest that a deeper understanding of local strain diversity through molecular typing could improve our knowledge of interspecies transmission dynamics and may help identify which animal reservoirs are most relevant to human Q fever in Saudi Arabia.

The high herd-level seroprevalence (e.g., 92% in goats) aligns with global reports on pastoral systems [25,26,30], but the lack of clinical data (e.g., abortion rates) precluded us from linking seropositivity to disease outcomes, a gap identified in similar studies [26,27,36]. Future work should integrate molecular tools such as PCR to detect active shedding of birth products/milk [8] and environmental sampling to evaluate the aerosol risks [6,12]. Despite these limitations, our findings provide the first national baseline for Saudi Arabia, informing One Health strategies to mitigate zoonotic transmission where human–animal contact is frequent [18]. Livestock exposure is a risk indicator, and species-specific contributions to human infections remain unclear through molecular and epidemiological linkage studies.

## 5. Conclusions

In conclusion, this nationwide study revealed a high seroprevalence of *Coxiella burnetii* among livestock in Saudi Arabia, with significant variations across regions and animal species. The results highlight the role of livestock—particularly small ruminants—as important reservoirs for Q fever, posing a clear zoonotic risk. This risk is especially elevated in areas with high densities of small ruminants, such as Jazan and Hail, as well as in peri-urban regions like Riyadh and Makkah.

The widespread exposure observed calls for integrated One Health strategies. These should include public health education on the dangers of consuming raw milk and close contact with livestock, along with strengthened surveillance and diagnostic efforts. Enhanced biosecurity measures, particularly on farms near urban centers, are also essential.

While this study provides critical baseline data, limitations—including its cross-sectional design and the lack of age-stratified or clinical data—warrant further longitudinal and molecular research to better understand transmission dynamics, identify active shedders, and evaluate the clinical impacts in both animals and humans.

## Figures and Tables

**Table 1:** Seroprevalence of *C. burnetii* in animals from different regions of Saudi Arabia.

Region	Sheep		Goats		Camels		Cattle		Cumulative	
	No. Positive/No. Tested (%)		No. Positive/No. Tested (%)		No. Positive/No. Tested (%)		No. Positive/No. Tested (%)		No. Positive/No. Tested (%)	
	Individuals	Herds	Individuals	Herds	Individuals	Herds	Individuals	Herds	Individuals	Herds
Hail	104/243 (42.8%)	33/45 (73.3%)	160/238 (67.2%)	41/45 (91%)	164/214 (76.6%)	42/45 (93.3%)	0/26 (0%)	0/6 (0%)	428/721 (59.3%)	116/141 (82.2%)
Riyadh	89/300 (29.7%)	48/59 (81.4%)	139/295 (47.1%)	48/59 (81.4%)	87/298 (29.2%)	57/60 (95%)	39/218 (17.9%)	20/46 (43.5%)	354/1111 (31.8%)	173/224 (77.2%)
Al-Sharqia	107/313 (34.2%)	48/55 (87.4%)	134/312 (42.9%)	52/55 (94.5%)	136/308 (44.2%)	47/55 (85.6%)	3/119 (2.5%)	3/20 (15%)	380/1052 (36.1%)	150/185 (81%)
Al-Qassim	71/180 (39.4%)	29/36 (87.3%)	73/160 (45.6%)	29/32 (90.6%)	125/180 (69.4%)	35/36 (97.2%)	3/92 (3.3%)	2/19 (10.5%)	272/612 (44.4%)	95/123 (77.2%)
N. Boundaries	29/145 (20%)	14/25 (87.3%)	34/142 (23.9%)	21/25 (84%)	71/144 (49.3%)	23/25 (92%)	1/36 (2.8%)	1/6 (16.7%)	135/467 (28.9%)	59/81 (72.8%)
Al-Gouf	51/149 (34.2%)	24/25 (96%)	80/139 (57.6%)	23/25 (92%)	55/157 (35%)	24/25 (96%)	6/148 (4%)	3/25 (12%)	192/593 (32.3%)	74/100 (74%)
Tabuk	39/200 (19.5%)	28/35 (80%)	90/207 (43.5%)	34/35 (97.1%)	124/201 (61.7)	34/35 (97.1%)	4/33 (12.1%)	3/6 (50%)	257/641 (40%)	99/111 (89.1%)
Gazan	21/60 (35%)	9/10 (90%)	28/57 (49.1%)	10/10 (100%)	18/57 (31.6%)	8/10 (80%)	2/60 (3.3%)	1/10 (10%)	69/234 (29.4%)	28/40 (70%)
Najran	5/29 (17.2%)	4/5 (80%)	10/26 (38.5%)	3/5 (60%)	15/26 (57.7%)	5/5 (100%)	-	-	30/81 (37%)	12/15 (80%)
Asir	32/150 (21.3%)	17/25 (68%)	70/148 (47.3%)	24/25 (96%)	67/145 (46.2%)	23/25 (92%)	4/145 (2.8%)	3/25 (12%)	173/588 (29.4%)	67/100 (67%)
Al-Baha	35/108 (32.4%)	16/19 (84.2%)	62/117 (53%)	18/20 (90%)	35/86 (40.7%)	14/15 (93.3%)	8/86 (9.3%)	6/15 (40%)	140/397 (35.2%)	54/69 (78.2%)
Madinah	47/173 (27.2%)	25/30 (83.3%)	96/177 (54.2%)	30/30 (100%)	82/170 (48.2%)	26/30 (86.7%)	5/36 (15.2%)	4/7 (57.1%)	230/556 (41.3%)	85/97 (87.6%)
Makkah	51/203 (25.1%)	36/45 (80%)	91/206 (44.2%)	45/45 (100%)	35/186 (18.8%)	44/45 (97.8%)	16/112 (14.2%)	14/32 (43.8%)	193/707 (27.2%)	139/167 (83.2%)
Total	681/2253 (30.2%)	331/414 (80%)	1067/2224 (48%)	378/411 (92%)	1014/2172 (46.7%)	382/411 (92.9%)	91/1111 (8.2%)	60/217 (27.6%)	2853/7760 (36.7%)	1151/1453 (79.2%)
*p*-Value	0.000 **	0.07 *	0.000 **	0.005 **	0.000 **	0.31 ^NS^	0.000 **	0.0014 **	0.001 **	0.003 **

NS: Non-significant difference (*p* > 0.05); * significant difference (*p*  <  0.05); **: highly significant difference (*p* ≤ 0.01).

## Data Availability

The data are contained within this manuscript.
